# A New HiPlex Amplicon Sequencing Approach for the Detection of Grapevine Leafroll-Associated Virus 3 and Grapevine Red Blotch Virus in Grapevines

**DOI:** 10.3390/v18090959

**Published:** 2026-09-01

**Authors:** Raied Abou Kubaa, Kristian A. Stevens, Teresa M. Erickson, Maher Al Rwahnih

**Affiliations:** 1 Foundation Plant Services, University of California, Davis, Davis, CA 95616, USA; raboukubaa@ucdavis.edu (R.A.K.); tmerickson@ucdavis.edu (T.M.E.); 2 Department of Plant Pathology, University of California, Davis, Davis, CA 95616, USA; 3 Department of Evolution and Ecology, University of California, Davis, Davis, CA 95616, USA; kastevens@ucdavis.edu; 4 Department of Plant Protection, School of Agriculture, The University of Jordan, Amman 11942, Jordan

**Keywords:** HiPlex PCR, grapevine viruses, GLRaV3, GRBV, virus diagnostics, high-throughput sequencing (HTS)

## Abstract

Grapevine leafroll-associated virus 3 (GLRaV3) and grapevine red blotch virus (GRBV) are two major pathogens associated with significant economic losses, requiring reliable and sensitive diagnostic tools for rapid identification. Current RT-qPCR and qPCR assays are widely used in certification programs; however, the risk of false-negative results due to the genetic diversity of plant viruses is always a concern. Here, we present a new targeted HiPlex assay designed for the detection of GLRaV3 and GRBV. A custom primer panel containing 98 oligonucleotides targeting GLRaV3 and 14 targeting GRBV was evaluated across 45 different grapevine accessions. HiPlex diagnostic performance metrics were evaluated up to the 1:1000 dilution level. The HiPlex assay detected both viruses in 30 ng of total nucleic acids (TNA) and at 1:10 and 1:100 dilutions, exhibiting a strong inverse correlation between read counts and Ct values. Analysis of reads from qPCR-negative samples allowed the determination of a positivity cutoff of 5000 reads, which provided high specificity while maintaining sensitivity. The results presented here support the application of HiPlex amplicon sequencing for the detection of GLRaV3 and GRBV and provides a foundation for future expansion of the primer panel to additional grapevine viruses. To our knowledge, this is the first study evaluating multiplex PCR-based amplicon sequencing specifically for grapevine virus diagnostics.

## 1. Introduction

Grapevine (*Vitis vinifera* L.) is one of the world’s most valuable perennial fruit crops, supporting major international industries for wine, table grapes, and raisins across diverse viticultural regions [1]. Viral infections represent a persistent constraint to grapevine productivity, with vegetative propagation contributing to the maintenance and dissemination of viruses through infected propagation material [2,3]. To date, 104 viral entities have been identified in grapevine, highlighting the remarkable diversity of its virome [4]. Among these, grapevine leafroll-associated virus 3 (GLRaV3) and grapevine red blotch virus (GRBV) are two of the most impactful pathogens affecting viticulture. GLRaV3 is widely distributed in grape-growing regions worldwide. Both viruses are economically important and have been linked to significant long-term economic losses. GRBV can reduce vineyard returns by up to $68,548 per hectare [5] while losses associated with GLRaV3 have been estimated at up to $41,000 per hectare over the lifespan of an affected vineyard [6]. In grapevine, RT-PCR based and real-time RT-PCR-based multiplexing assays have been used for the simultaneous detection of multiple viruses [7,8]. Although this approach improved diagnostic efficiency, the ability to detect multiple virus targets in conventional or real-time PCR assays is constrained by the need for each end point PCR product to be distinguishable by size or each real-time assay to have a unique reporter dye. The optimization of multiplex PCR assays is complicated due to primer interactions and reduced PCR efficiency as the number of primers increases. Multiplex amplification-based sequencing techniques (HiPlex PCR) have been initially applied in both human and animal studies [9], and were subsequently adapted for plant virus diagnostics [10,11]. HiPlex PCR enables the simultaneous use of a larger number of primer pairs, all producing short amplicons, followed by high throughput sequencing to confirm amplicon identity. GLRaV3 and GRBV were chosen as targets for this study due to both their economic significance and their differences in genome types. Although several diagnostic techniques have been developed for the detection of GLRaV3 and GRBV, these two viruses provide suitable targets for evaluating the HiPlex amplicon sequencing approach. In particular, GLRaV3 exhibits extensive genetic diversity across multiple phylogenetic groups [12,13], making it a suitable target for evaluating a multiplex amplicon sequencing approach designed to incorporate multiple primer pairs targeting diverse viral variants.

This need aligns with recent national diagnostic advancements, as U.S. regulatory agencies have formally adopted high-throughput sequencing (HTS) together with qPCR-based assays as approved methods for quarantine and certification testing [14], further emphasizing the growing role of sequencing-based diagnostics in grapevine health programs. Although HTS has become important for grapevine virus detection, it is typically used to establish initial phytosanitary status of small quantities of source material, while laboratories conducting routine testing to verify health of propagated plant material and vineyards require targeted methods that are more adaptable to large-scale screening workflows. In the present study, we developed and evaluated a HiPlex amplicon sequencing assay for the detection of GLRaV3 and GRBV, using a primer panel designed to capture the genetic diversity of both viruses. The assay was evaluated using grapevine accessions from the Foundation Plant Services (FPS) collection at the University of California Davis, representing different infection profiles for GLRaV3 and GRBV, including single infections, mixed infections, and virus-negative controls. Performance of the assay was assessed across serial dilutions and compared with established qPCR assays to evaluate sensitivity and specificity.

This objective is particularly relevant to the grapevine industry, where routine testing of foundation and certification material is essential for maintaining clean planting stock and reducing the risk of virus dissemination through propagation. Compared with conventional qPCR workflows that typically require multiple independent assays and metagenomic HTS approaches that distribute sequencing depth across host and non-target nucleic acids, HiPlex provides a targeted and scalable strategy capable of simultaneously screening large numbers of samples and viral targets within a single sequencing run. Previous HiPlex studies demonstrated the simultaneous detection of up to 34 viruses and viroids across more than 100 fruit tree samples in a single sequencing run [11], highlighting the potential scalability of this approach for certification programs and large-scale screening workflows. To our knowledge, this study represents the first application and evaluation of multiplex PCR-based amplicon sequencing specifically for grapevine virus diagnostics. We hypothesized that HiPlex amplicon sequencing using multiple virus-specific primer pairs would reliably detect GLRaV3 and GRBV across the grapevine accessions evaluated in this study and show agreement with established qPCR assays.

## 2. Materials and Methods

### 2.1. Plant Material and TNA Extraction

A total of 45 grapevine accessions were included in the study, representing GLRaV3-positives, GRBV-positives, mixed infections with the two viruses, and control plants free from both viruses (Table 1). All plant material was obtained from the Foundation Plant Services (FPS) grapevine collection at the University of California, Davis. The phytosanitary status of each accession was based on historical FPS diagnostic records generated using validated virus-testing protocols routinely implemented in certification and quarantine programs. Fresh grapevine tissue (approximately 0.8 g per sample) was homogenized in 8 mL of guanidine isothiocyanate lysis buffer (4 M guanidine isothiocyanate; 0.2 M sodium acetate, pH 5.0; 2 mM EDTA; 2.5% (*w*/*v*) PVP-40) and used for total nucleic acids (TNAs) extraction. TNAs used in this study were collected from leaf petioles or bark scrapings, depending on the available plant material. The study also included previously extracted TNAs from archived grapevine accessions maintained at FPS.

TNAs were extracted using the MagMAX Plant RNA Isolation Kit (ThermoFisher Scientific, Waltham, MA, USA) following the manufacturer’s instructions and omitting the DNase step. Extracted TNAs were quantified using the Qubit^TM^ RNA BR Assay Kit (ThermoFisher Scientific, Waltham, MA, USA). All samples were brought to an initial 30 ng/uL starting concentration by dilution with water if needed. Common bean (*Phaseolus vulgaris*) cultivar Black Turtle Soup (BTS), containing the persistent endornaviruses, Phaseolus vulgaris endornavirus 1 (PvEV-1) and PvEV-2, was included as an internal amplification control in the assay [15]. During sample preparation, 5% (*w*/*w*) BTS leaf material was processed with the grapevine petiole samples. Additional BTS control dilutions were included to assess the effect of internal-control concentration on sequencing output at low template levels. Some samples were processed without BTS spike-in to assess assay performance in the absence of an internal control. To evaluate the sensitivity of the assay for virus detection, four ten-fold dilutions (1:10, 1:100, 1:1000 and 1:10,000) were prepared from each 30 ng/µL normalized sample (NS) and used for cDNA synthesis. The dilution series was included to evaluate the practical detection range of the assay and to assess the relationship between HiPlex read counts and qPCR Ct values across decreasing template concentrations.

### 2.2. Dilution Series and cDNA Synthesis

First-strand cDNA was generated from 10 µL of the NS (30 ng/µL) and the four dilutions, using the Maxima H Minus First Strand cDNA Synthesis Kit (Thermo Scientific, Waltham, MA, USA) according to the manufacturer’s protocol. The final reaction volume was 25 µL. Each reaction contained 10 µL of template, 1.25 µL of random hexamers (100 pmol), and 1.25 µL of dNTPs mix. cDNA reactions were prepared in Bio-Rad Hard-Shell^®^ 96-well PCR plates (Bio-Rad Laboratories, Inc., Hercules, CA, USA). Three technical replicates were prepared for each dilution, along with two types of controls: a non-template control (NTC) and a healthy grapevine control processed in parallel with all samples. To monitor potential contamination during plate preparation and downstream processing, two separate rows of NTCs were included in each plate, with one added before template loading and the other added after cDNA preparation. In total, 675 cDNA reactions were generated in this study, and 312 water controls were included.

### 2.3. Primer Design

Publicly available full-genome sequences, and internal FPS sequence data for GLRaV3 and GRBV were aligned to identify regions of conserved sequences suitable for primer design. Alignment and primer design were performed using Geneious Prime software version 2024.0.2 (Biomatters Ltd., Auckland, New Zealand; https://www.geneious.com/), following the general framework established in earlier HiPlex implementations. Target amplicon lengths were restricted to 90–110 bp to ensure consistent amplification and sequencing efficiency. Because GLRaV3 exhibits substantial genetic diversity across multiple phylogenetic groups [12,13], all available isolates from GenBank and the internal database from FPS were clustered into closely related sequence groups, and separate primer sets were designed for each cluster to ensure broad representation across variants. The final HiPlex panel evaluated in this study included 49 GLRaV3 amplicon targets, seven GRBV amplicon targets, and ten internal control primer pairs targeting PvEV-1 and PvEV-2, (Supplementary Table S1). The primer set included multiple group-specific primers designed to improve coverage across divergent GLRaV3 variants and GRBV isolates. For both viruses, the amplicon targets were distributed across the viral genomes, covering multiple coding regions rather than being restricted to a single gene or genomic region. Primer specificity and potential cross-reactivity were evaluated in silico using the ThermoFisher Multiple Primer Analyzer (ThermoFisher Scientific, Waltham, MA, USA) and NCBI Primer-BLAST (https://www.ncbi.nlm.nih.gov/). The final multiplex panel contained 132 primers (Supplementary Table S1). All primers pairs were synthesized by Integrated DNA technologies (Coralville, IA, USA). Additional primer sets targeting other grapevine viruses were included but not evaluated in this study. The presence of primers targeting other viruses provided us the opportunity to assess potential cross-reactivity and non-specific amplification with GLRaV3 and GRBV within the highly multiplexed HiPlex framework.

### 2.4. HiPlex Protocol

Following extraction, HiPlex amplification was performed using the optimized multiplex protocol implemented at Floodlight Genomics (Knoxville, TN, USA) and according to [9,11]. All primers were assembled into a single multiplex pool, which was combined with 2 µL of cDNA template and the proprietary adaptor mix required for downstream barcoding. Following PCR amplification, the full set of amplicons generated from each sample was treated as a single barcoded pool for subsequent processing. Before library preparation, amplicons were purified using the QIAquick Gel Extraction Kit (Qiagen, Hilden, Germany) for size selection, and the resulting purified products were quantified with the Qubit 1× HS dsDNA Assay Kit (Invitrogen, Carlsbad, CA, USA). Library preparation for the pooled barcoded amplicons was performed using the KAPA HyperPrep Kit (PCR-free) (Roche Molecular Systems, Rotkreuz, Switzerland) following the manufacturer’s instructions. Library quality and fragment size distribution were analyzed using TapeStation 4200 (Agilent, Santa Clara, CA, USA), and final DNA concentration was measured using the KAPA Library Quant Kit (Illumina/ROX Low) (Roche, Diagnostics, Indianapolis, IN, USA). The completed library was sequenced in paired-end mode on an Illumina NextSeq 2000 platform (San Diego, CA, USA) using a P1 reagent kit (300 cycles). A 30% PhiX spike-in was included to ensure balanced sequencing diversity, and the final loaded concentration of the library was adjusted to 0.8 nM. Bioinformatic processing followed the streamlined HiPlex workflow previously described [10,11] with adaptations for the grapevine-specific primer panel. After demultiplexing and quality filtering, sequencing reads were then mapped to a small database of curated viral reference sequences (Supplementary Table S6); 11 genomes of GLRaV3, 3 genomes of GRBV, and the positive control genomes for PvEV-1 and PvEV-2, using bowtie2 (v2.5) [16] as implemented in [17]. The read counts reported for each sample were obtained by summing the number of distinct reads mapping to all references for each distinct virus in the assay.

For each extraction and dilution, amplification and sequencing were performed twice for all 3 replicates. This created a total of six technical replicates for each sample. The average mapped read count across the available technical replicates was used to determine the final sample-level HiPlex result and for all downstream diagnostic performance analyses. Replicate-level data are provided in the Supplementary Tables S3–S5). A sample was considered positive when the average read count exceeded the empirical threshold of 5000 mapped reads. Replicates with no data were considered 0 for the purpose of this average.

To determine the read count threshold by which positives were called, read counts for all replicates were collated to empirically establish a detection threshold for all viruses in the assay. For each sample and dilution level, technical replicates were analyzed independently.

### 2.5. RT-qPCR

The same 45 TNA extracts used for HiPlex amplification, including the NS (30 ng/µL) and all dilution levels, were also analyzed by RT-qPCR and qPCR using validated assays for GLRaV3 and GRBV. Analyses were performed to confirm the phytosanitary status of each accession prior to HiPlex evaluation and to monitor changes in target detection across the serial dilution series. Primer and probe sequences, as well as reaction conditions, followed previously validated protocols routinely implemented at FPS as described in [12,18]. Although routine FPS diagnostic decisions are not based on averaging technical replicates, each sample was analyzed in duplicate qPCR reactions. In this study, and for comparison with the HiPlex workflow, the mean Ct value from the two technical replicates was used for all downstream analyses. The Ct values were then correlated with HiPlex sequencing read counts to assess the association between target concentration and read abundance.

## 3. Results

### 3.1. HiPlex Sequencing Performance Across Dilution Levels

Sequencing of the pooled HiPlex amplicons generated from all grapevine samples (NS and dilution levels) produced approximately 518 million raw reads on the Illumina NextSeq 2000 platform. The HiPlex panel evaluated in this study included 49 amplicon targets for GLRaV3 and seven amplicon targets for GRBV. HiPlex amplicon sequencing generated consistent and detectable read counts across the evaluated dilution series for both GLRaV3 and GRBV. Strong amplification signals were observed for NS, 1:10, and 1:100 samples, whereas the 1:1000 dilution produced lower but still detectable read counts. Dilutions beyond 1:1000 generally fell below the practical detection range of the assay and were excluded from subsequent analyses. These observations indicate that the current HiPlex workflow maintains reliable detection performance primarily within the NS to 1:100 dilution range under the experimental conditions evaluated in this study.

### 3.2. Empirical Determination of the HiPlex Positivity Cutoff

**Figure 1:**
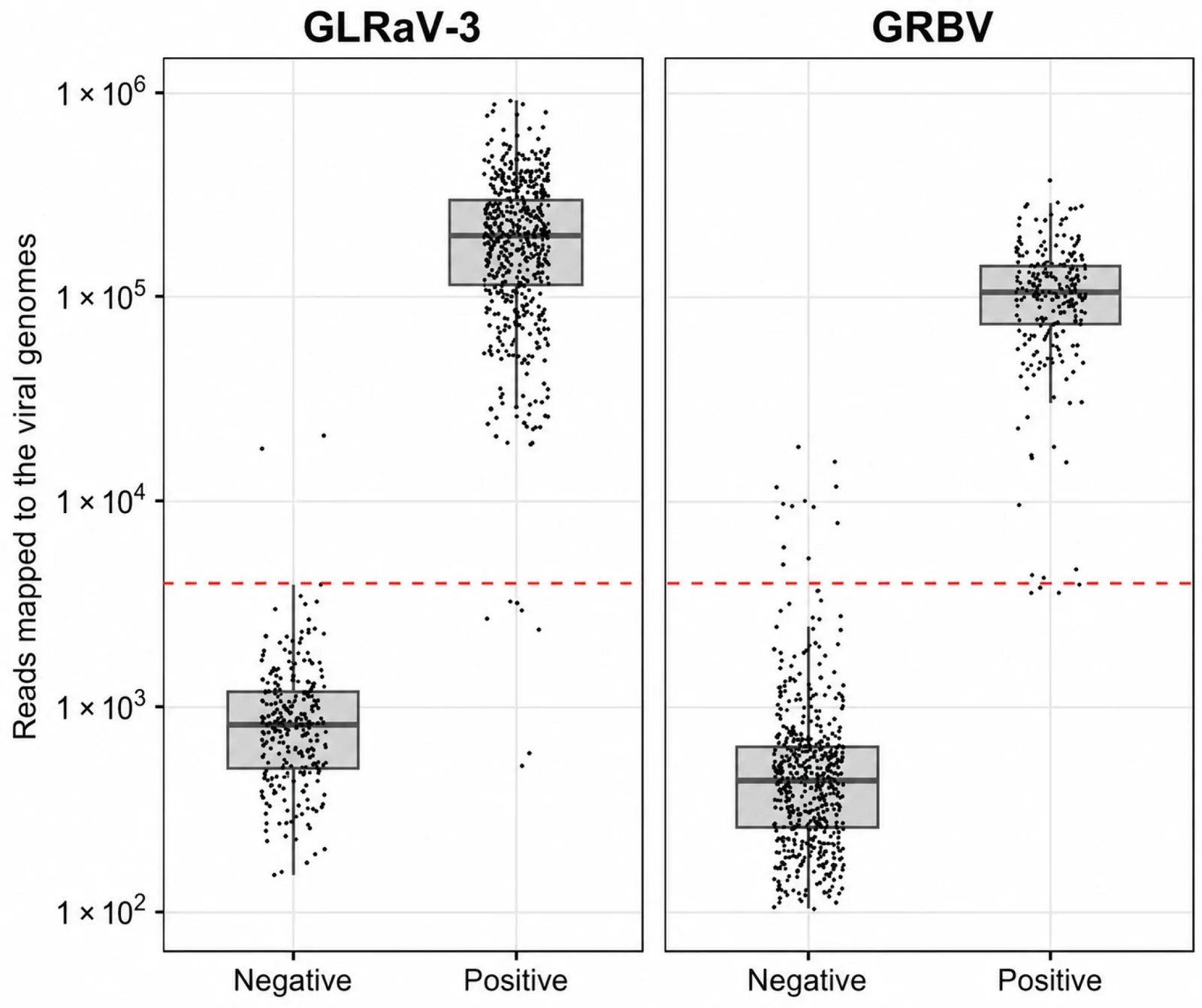
Total number of HiPlex mapped reads for the expected (true positive) and non-expected (false positive) detections of GLRaV3 (**left**) and GRBV (**right**) across all evaluated samples, technical replicates, and dilution levels included in this study. Each point represents a single technical replicate. The red dashed line indicates the established cutoff used to classify samples as positive.

For both GLRaV3 and GRBV, an empirical background threshold of 5000 mapped HiPlex reads per sample was established based on the distribution of read counts observed in qPCR-negative samples and all associated technical replicates included in this study (Figure 1). Most qPCR-negative samples across the evaluated dilution series produced fewer than 2000 mapped reads, whereas only a limited number of outliers exceeded the 5000-read threshold. These elevated values were generally inconsistent across replicates and were primarily observed in some diluted templates. No water control well produced virus-specific reads except for a single GRBV-associated event. Out of 312 water control wells included in this study, only 1 (0.32%) generated a high number of GRBV-associated reads, whereas GLRaV3 and the internal control targets PvEV-1 and PvEV-2 remained below the positivity threshold in the same well. Because this event was target-specific, non-reproducible, and not accompanied by amplification of other targets, it was interpreted as a sporadic carryover event most likely associated with sample handling or plate processing rather than systematic contamination. Similar sporadic background amplification patterns have previously been reported in HiPlex amplicon sequencing assays [10]. Based on these observations, 5000 mapped reads were considered an appropriate threshold to distinguish true positive detections from background amplification under the current assay configuration and sequencing conditions.

### 3.3. Agreement Between HiPlex Sequencing and qPCR Detection

All grapevine samples previously characterized as positive for GLRaV3 and/or GRBV were consistently detected by the HiPlex assay at NS, 1:10, and 1:100, whereas detection became more variable at the highest dilution levels, particularly for samples with high Ct values (Supplementary Tables S3 and S4). Virus-negative grapevine controls remained below the empirical background threshold established for the assay. Internal controls targeting PvEV-1 and PvEV-2 produced strong read counts in samples and control dilutions where BTS material was included, confirming successful amplification and sequencing when sufficient internal-control template was present. In samples where BTS was absent or highly diluted, internal-control read counts were reduced or absent, highlighting the importance of including an internal control at a consistent concentration across dilution levels (Supplementary Table S5).

To evaluate the effect of template dilution on HiPlex detection, representative GLRaV3- and GRBV-positive grapevine samples were analyzed across serial dilutions. As expected, progressive dilution resulted in a reduction in HiPlex mapped read counts accompanied by increasing qPCR Ct values (Supplementary Figure S1), demonstrating agreement between HiPlex sequencing output and qPCR-derived estimates of viral titer. To further examine the relationship between RT-qPCR and HiPlex sequencing results, samples were grouped into Ct-value classes and the distribution of HiPlex mapped reads was evaluated within each category (Supplementary Figure S2). A progressive decline in mapped read counts was observed with increasing Ct values, indicating reduced viral template abundance and lower sequencing signal at higher Ct ranges. Samples within the 15–25 Ct classes consistently produced the highest read counts, whereas samples in the 31–34 and 35–40 Ct classes showed substantially lower read numbers and a higher proportion of zero-read observations. Most zero-read detections in the higher Ct classes originated from highly diluted templates (with positive qPCR at first dilutions), particularly the 1:1000 dilution level, reflecting the expected reduction in assay sensitivity near the practical detection limit. Overall, detections above the 5000-read threshold were predominantly associated with Ct values below 30. Samples with Ct values between 31 and 35 showed a transition zone, with most GRBV detections falling below the threshold, whereas a small number of GLRaV3 samples remained above it. At Ct values between 36 and 40, HiPlex read counts were generally below the positivity threshold for both viruses, with only rare exceptions. Replicate-level read counts for all samples and dilution levels are provided in Supplementary Tables S3–S5. For downstream diagnostic performance analyses, positivity calls were based on the average mapped read count obtained from the available technical replicates for each sample and dilution level.

### 3.4. Diagnostic Performance

Using the empirical threshold of 5000 mapped reads, the diagnostic performance of the HiPlex workflow was evaluated relative to corresponding qPCR results across all dilution levels (Table 2). Sensitivity and specificity were calculated as TP/(TP + FN) and TN/(TN + FP), respectively, where TP, TN, FP, and FN represent true positives, true negatives, false positives, and false negatives [19,20]. Positive calls were based on the average mapped read count obtained across the available technical replicates for each sample and dilution level. For both GLRaV3 and GRBV, NS and the 1:10 and 1:100 dilution levels showed high agreement between HiPlex and qPCR results, with sensitivity values reaching 100% for both viruses. At the 1:1000 dilution level, sensitivity decreased substantially, particularly for GRBV, reflecting the lower analytical sensitivity observed at extreme template dilutions. This reduction was primarily driven by samples with high Ct values and highly diluted templates rather than by failures in samples with moderate or high viral titers. Specificity remained consistently high across all evaluated conditions, with only a single anomalous GRBV signal exceeding the empirical threshold in 2 of 3 technical replicates. Similar to observations reported by [11], this isolated event was not reproducible across the remaining replicates or dilution levels of the same sample and was therefore considered more consistent with background noise or sporadic carryover than with true positive detection.

## 4. Discussion

As expected, amplification dynamics used in highly multiplexed amplicon-based analysis can affect the number of reads recovered for each viral target. Consequently, the detection thresholds established in this study take into account the current panel design, and any modifications to the design, e.g., exclusion or inclusion of certain targets, would also have consequences for the read depth distribution and, possibly, empirical thresholds. In addition, the total number of samples and the proportion of virus-positive samples in the sequencing library may also affect read depth distribution. The qPCR negative samples produced a few high reads but generally not in all technical replicates. In this regard, the sporadic signals observed in this study may indicate occasional carry-over contamination during laboratory procedures. Indeed, similar signals were occasionally observed in other highly multiplexed HiPlex studies [10]. In routine diagnostics, samples generating late qPCR signals would typically be retested to exclude sporadic contamination events. In this study, although some historically negative samples produced such borderline qPCR signals at the original concentration, the corresponding HiPlex read counts remained below the 5000-read threshold. These observations support their classification as negative and further demonstrate the robustness of the HiPlex workflow when interpreting occasional borderline qPCR signals. They also emphasize the importance of evaluating technical replicates when interpreting isolated high read counts. This was particularly evident in several borderline observations where isolated signals were not reproduced across the remaining technical replicates of the same sample and dilution level.

High-titer and high-concentration samples yielded relatively strong and consistent HiPlex signals, whereas borderline samples with high Ct or extreme dilution levels showed low and variable read counts. The Ct-class analysis further demonstrated a clear inverse relationship between qPCR Ct values and HiPlex mapped reads, while the few exceptions observed at higher Ct values were primarily associated with highly diluted templates near the practical assay detection limit. This observation was further supported by the qPCR performance analysis, which showed that detectable qPCR signals persisted at dilution levels where HiPlex sensitivity was substantially reduced.

These findings indicate that HiPlex achieves diagnostic performance comparable to qPCR under routine and moderate dilution conditions, with sensitivity decreasing primarily at extreme dilution levels near the practical assay detection limit. The isolated GRBV-positive signal observed in one water control well was excluded from the diagnostic performance calculations because it did not correspond to a biological sample and was not accompanied by amplification of other viral targets.

Isolated signals also support the importance of considering technical replicates to analyze the data from highly multiplexed workflows and samples near the detection threshold. The results obtained in this study further support the use of technical replicates when applying highly multiplexed HiPlex assays, particularly for samples near the practical detection limit. Similar behavior was observed in previous studies, with low-titer samples and highly diluted samples showing increased signal variability and lack of consistency [10,11]. The internal-control results (Supplementary Table S5) further suggest that maintaining a consistent concentration of control template across dilution levels, as previously implemented by [10] may improve interpretation of low-titer samples and facilitate differentiation between true amplification failure and reduced signal associated with samples near the practical detection limit. The high congruency observed between qPCR and HiPlex results for non-borderline and moderately diluted samples further demonstrated the efficacy of the targeted amplicon sequencing methodology for grapevine virus testing.

New diagnostic technologies require formal validation before routine implementation, especially for use on plant material regulated by quarantine and/or certification programs. Therefore, this study was designed to generate performance data supporting future certification and regulatory applications of the HiPlex workflow, similar to previous HTS validation efforts [15]. Over time, metagenomic HTS has played a key role in the discovery and classification of novel viruses [21,22,23,24,25]. Recently, HTS technologies have evolved from virus discovery approaches into powerful diagnostic platforms capable of supporting virus quarantine, certification, and clean plant initiatives [14,17,26]. The adoption of HTS approaches within grapevine certification and clean plant programs has greatly improved the ability to detect mixed infections and divergent variants in cases where conventional testing failed. In addition, the application of HTS diagnostics within the field of virus diagnosis has led to a significant improvement in the understanding of plant viromes. However, metagenomic approaches typically allocate a large proportion of sequencing reads to host-derived nucleic acids, which reduces analytical efficiency for routine diagnostics [27,28]. In this diagnostic transition, targeted amplicon approaches such as HiPlex provide even more scalability because sequencing reads can be concentrated toward target viruses instead of being equally distributed in all samples. The HiPlex amplicon-based sequencing concentrates all sequencing depth toward predefined targets, which may provide advantages for targeted virus detection compared with untargeted sequencing approaches. As shown in this study, virus-derived reads can be efficiently recovered using the HiPlex assay. Because sequencing depth in highly-multiplexed workflows is shared among all viral targets in the reaction mixture, a targeted approach such as HiPlex may substantially improve the efficiency of the test. This is an added value of HiPlex with respect to untargeted total RNA sequencing which requires greater sequencing depth. This enrichment effect has also been recognized as one of the key advantages of targeted HTS approaches in both human or animal viruses [9,29,30,31].

As shown in this study, the application of HiPlex amplicon sequencing to diagnose grapevine viruses is feasible when using GLRaV3 and GRBV as target viruses. Considering the high degree of genetic variability found in several grapevine viruses, in particular GLRaV3 [12,13], it was necessary to include multiple primer pairs in the same HiPlex panel to increase detection specificity within multiplex reactions. Despite the extensive genetic diversity of GLRaV3, the multi-target primer design strategy provided consistent detection across all GLRaV3-positive accessions included in this study. A great strength of the HiPlex assay lies in the modularity of the multiplex panel itself. New primer pairs can be added, optimized, or excluded from the workflow when new targets become relevant to certification protocols. Thus, the modularity of the HiPlex assay allows for future incorporation and optimization of other grapevine viral targets. This work establishes a foundational framework for the application of HiPlex amplicon sequencing to GLRaV3 and GRBV detection and for evaluating its potential future expansion to additional regulated grapevine viruses, particularly genetically diverse viruses such as members of the GLRaV4 complex, where broad variant coverage remains important for reliable detection. Based on the results of the present study, efforts have been initiated to expand and validate the HiPlex platform for the detection of other regulated grapevine viruses relevant to the California grapevine industry. As a result, a comprehensive HiPlex workflow is being developed for grapevine virus testing. In summary, this work shows that HiPlex amplicon sequencing can provide a scalable and sequence-informed approach to grapevine diagnostics while supporting future expansion towards regulated virus panels. Further validation with expanded virus panels and larger sample sets will be needed to evaluate the applicability of this workflow to routine certification and clean plant programs.

## Figures and Tables

**Table 1:** Grapevine accessions included in this study and their corresponding infection status with GLRaV3 and GRBV.

N	FPS Code	Cultivar	GLRaV3	GRBV
1	GVX-01-L984	Chardonnay	+	+
2	GVX-02-L985	Cabernet Sauvignon	+	+
3	GVX-03-L986	Princess	+	+
4	GVX-04-L987	W267	−	+
5	GVX-05-L988	Chardonnay	+	+
6	GVX-06-L989	Chardonnay	+	+
7	GVX-07-L990	Private selection	+	−
8	GVX-08-L991	Dk #03	+	−
9	GVX-09-L992	Planta Fina	−	+
10	GVX-10-L993	Private selection	+	−
11	GVX-11-L994	Ill.172-7	+	+
12	GVX-12-L995	Babica	+	−
13	GVX-13-L996	Nero d’Avola	+	−
14	GVX-14-L997	Eftakilo	+	−
15	GVX-15-L998	Black Corinth	+	−
16	GVX-16-L999	Green Veltliner	+	−
17	GVX-17-M000	Marzemino	+	−
18	GVX-18-M001	Mladenka	+	−
19	GVX-20-M003	Charbono	+	+
20	GVX-22-M004	Nakachevanin Sahibi	−	−
21	GVX-23-N345	Gechi Kyrlen	−	−
22	GVX-24-N346	Ekdona Turkmenskaya	−	−
23	GVX-25-N342	Catarratto	+	−
24	GVX-26-N347	Catarratto	+	−
25	GVX-27-N332	Cortese	+	+
26	GVX-28-N333	Midget Thompson Seedless	−	−
27	GVX-29-N334	Seedless Emperor	+	−
28	GVX-30-N335	Fayoumi	+	−
29	GVX-31-N337	Thompson Seedless	−	−
30	GVX-32-N338	Black Seedless	−	−
31	GVX-33-N339	Zante Currant	+	−
32	GVX-34-N344	W070	−	+
33	GVX-39-N348	Zinfandel	−	+
34	GVX-40-N349	Zinfandel	−	+
35	GVX-41-N360	Tamar	+	−
36	GVX-42-N358	Shami	+	−
37	GVX-43-N359	Dk S Opushonnym Listom	−	−
38	GVX-44-N356	Kara Uzyum Nuhurski	−	−
39	GVX-45-N357	Nagano Purple	+	−
40	GVX-46-N361	Mavrodaphne	+	−
41	GVX-48-N350	Cabernet Sauvignon	+	−
42	GVX-49-N352	FLK101	−	−
43	GVX-50-N351	Seeded Thompson	−	−
44	GVX-51-N353	Tinta Miuda	−	−
45	GVX-52-N355	Private selection	+	−

**Table 2:** Positive and negative classifications were based on the average mapped read counts across the available technical replicates for each sample and dilution level. Sensitivity and specificity were then calculated from the final sample classifications. TP, true positive; FN, false negative; FP, false positive; TN, true negative.

Virus	Dilution	HiPlex					
		TP	FN	FP	TN	Sensitivity (%)	Specificity (%)
GLRaV3	NS	58	0	2	30	100	94
	1:10	58	0	0	32	100	100
	1:100	58	0	0	32	100	100
	1:1000	20	38	0	32	34	100
GRBV	NS	26	0	0	64	100	100
	1:10	26	0	1	63	100	96.9
	1:100	26	0	0	64	100	100
	1:1000	4	22	0	64	15.4	100

## Data Availability

Restrictions apply to the datasets.

## Supplementary Materials

The following supporting information can be downloaded at: https://www.mdpi.com/article/10.3390/v18090959/s1, Table S1. List of all primers included in the Hiplex reported in this study for GLRaV3 and GRBV; Table S2. Total number of filtered reads (≥100 bp) per sample generated by the amplicon sequencing; Table S3. Detection of the GLRaV3 (concentrated and diluted) by amplicon sequencing and individual RT-qPCR; Table S4. Detection of the GRBV (concentrated and diluted) by amplicon sequencing and individual qPCR; Table S5. Total read counts mapped to PvEV-1 and PvEV-2 (concentrated and diluted) generated by amplicon sequencing in each sample; Table S6. Curated viral reference sequences for GLRaV3 and GRBV; Figure S1. Comparison of average HiPlex mapped read counts and average qPCR Ct values across serial dilutions for representative grapevine samples infected with GLRaV-3 and/or GRBV; Figure S2. Relationship between qPCR Ct value classes and average HiPlex mapped reads for GLRaV-3 and GRBV.
