# Supplementary material for: A New HiPlex Amplicon Sequencing Approach for the Detection of Grapevine Leafroll-Associated Virus 3 and Grapevine Red Blotch Virus in Grapevines
Source: Viruses. 2026 Sep 1;18(9):959. doi: 10.3390/v18090959 (PMC13611930; doi:10.3390/v18090959)
Supplement: Supplementary file 1 [file viruses-18-00959-s001.zip › Supplimentary Figures Abou Kubaa.pdf]

# A New HiPlex Amplicon Sequencing Approach for Detection of Grapevine leafroll-associated virus 3 (GLRaV-3) and Grapevine red blotch virus (GRBV) in Grapevines

Raied Abou Kubaa[1,2], Kristian A. Stevens[1,3], Teresa M. Erickson[1], Maher Al Rwahnih[1,4]

*[1]Foundation Plant Services, University of California, Davis, Davis CA 95616, USA, [2]Department of Plant Pathology, University of California, Davis, Davis CA, 95616, USA, [3]Department of Evolution and Ecology, University of California, Davis, Davis, CA 95616, USA, [4]Department of Plant Protection, School of Agriculture, The University of Jordan, Amman, Jordan,*

## Supplementary figures

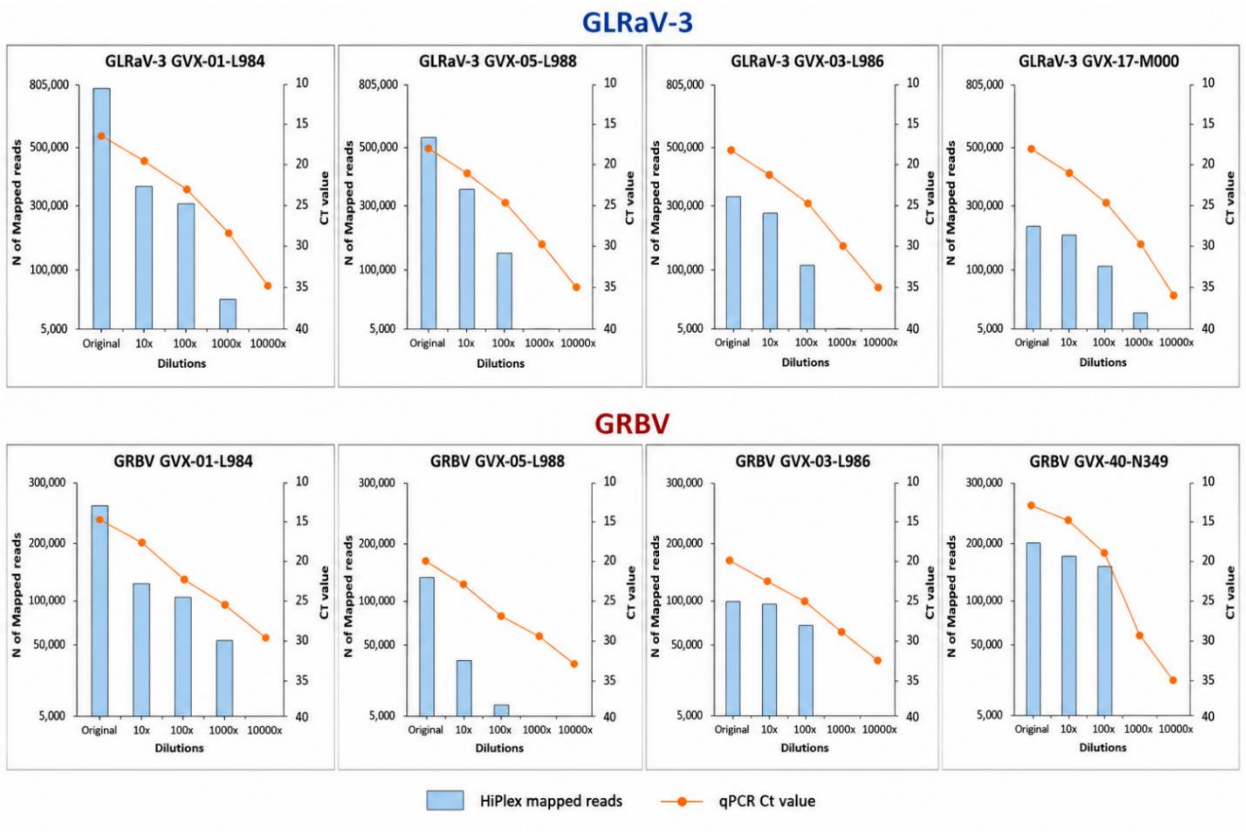


**Supplementary Figure S1.** Comparison of average HiPlex mapped read counts and average qPCR Ct values across serial dilutions for representative grapevine samples infected with GLRaV-3 and/or GRBV. Bars represent the mean HiPlex mapped reads across available technical replicates, and lines represent the mean qPCR Ct values across available technical replicates for the same sample and dilution level.

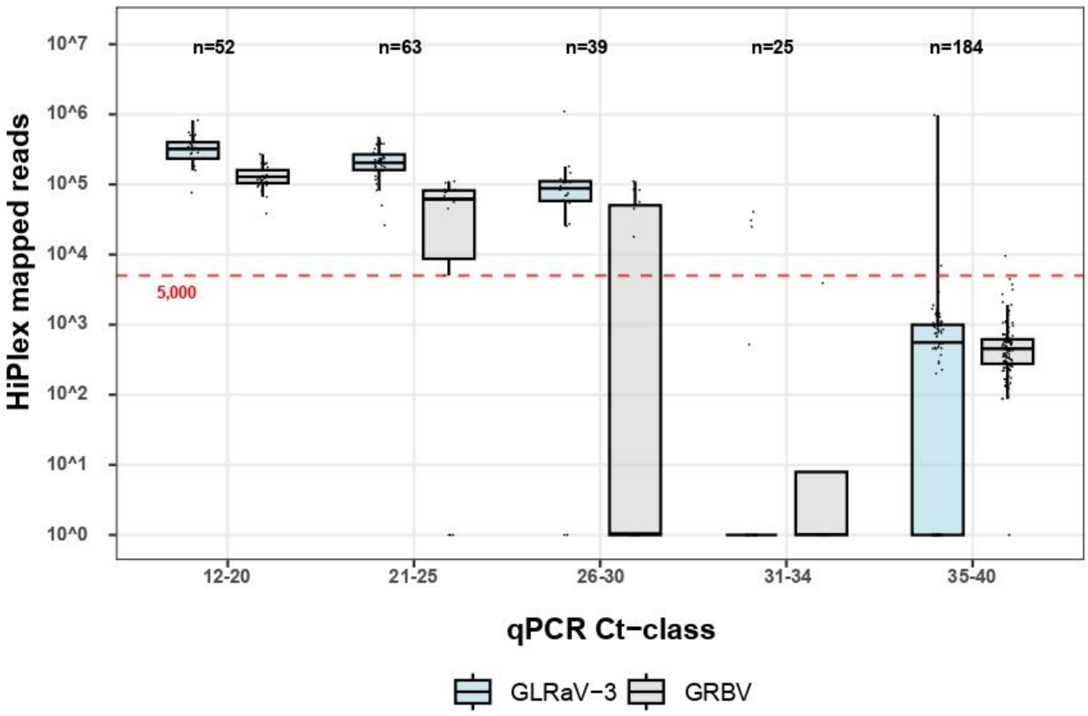


**Supplementary Figure S2.** Relationship between qPCR Ct value classes and average HiPlex mapped reads for GLRaV-3 and GRBV. Boxplots represent the distribution of average mapped reads within each Ct-value class, and dots represent sample-level average mapped read counts calculated from the available technical replicates. The red dashed line indicates the empirical positivity threshold of 5,000 mapped reads. Sample sizes are shown above each boxplot. qPCR non-detects were assigned a Ct value of 40, while zero mapped read counts were plotted as 1 read for visualization on the logarithmic scale. Consequently, the 35–40 Ct class includes both highly diluted positive samples and qPCR non-detects, resulting in a broader distribution of HiPlex mapped reads than the intermediate Ct classes.
